# In-office, in-home, and telehealth cognitive processing therapy for posttraumatic stress disorder in veterans: a randomized clinical trial

**DOI:** 10.1186/s12888-022-03699-4

**Published:** 2022-01-17

**Authors:** Alan L. Peterson, Jim Mintz, John C. Moring, Casey L. Straud, Stacey Young-McCaughan, Cindy A. McGeary, Donald D. McGeary, Brett T. Litz, Dawn I. Velligan, Alexandra Macdonald, Emma Mata-Galan, Stephen L. Holliday, Kirsten H. Dillon, John D. Roache, Lindsay M. Bira, Paul S. Nabity, Elisa M. Medellin, Willie J. Hale, Patricia A. Resick

**Affiliations:** 1 Department of Psychiatry and Behavioral Sciences, University of Texas Health Science Center at San Antonio, 7703 Floyd Curl Dr, San Antonio, TX 78229 USA; 2 South Texas Veterans Health Care System, San Antonio, TX USA; 3 Department of Psychology, University of Texas at San Antonio, San Antonio, TX USA; 4 Department of Rehabilitation Medicine, University of Texas Health Science Center at San Antonio, San Antonio, TX USA; 5 Department of Psychiatry, Boston University School of Medicine, Boston, MA USA; 6 Massachusetts Veterans Epidemiological Research and Information Center, VA Boston Healthcare System, Boston, MA USA; 7 Department of Psychological and Brain Sciences, Boston University, Boston, MA USA; 8 The Citadel, Military College of South Carolina, Charleston, SC USA; 9 Durham VA Health Care System, Durham, North Carolina USA; 10 Department of Psychiatry and Behavioral Sciences, Duke Health, Durham, North Carolina USA

## Abstract

**Background:**

Trauma-focused psychotherapies for combat-related posttraumatic stress disorder (PTSD) in military veterans are efficacious, but there are many barriers to receiving treatment. The objective of this study was to determine if cognitive processing therapy (CPT) for PTSD among active duty military personnel and veterans would result in increased acceptability, fewer dropouts, and better outcomes when delivered In-Home or by Telehealth as compared to In-Office treatment.

**Methods:**

The trial used an equipoise-stratified randomization design in which participants (*N* = 120) could decline none or any 1 arm of the study and were then randomized equally to 1 of the remaining arms. Therapists delivered CPT in 12 sessions lasting 60-min each. Self-reported PTSD symptoms on the PTSD Checklist for *DSM-5* (PCL-5) served as the primary outcome.

**Results:**

Over half of the participants (57%) declined 1 treatment arm. Telehealth was the most acceptable and least often refused delivery format (17%), followed by In-Office (29%), and In-Home (54%); these differences were significant (*p* = 0.0008). Significant reductions in PTSD symptoms occurred with all treatment formats (*p* < .0001). Improvement on the PCL-5 was about twice as large in the In-Home (d = 2.1) and Telehealth (d = 2.0) formats than In-Office (d = 1.3); those differences were statistically large and significant (d = 0.8, 0.7 and *p* = 0.009, 0.014, respectively). There were no significant differences between In-Home and Telehealth outcomes (*p* = 0.77, d = −.08). Dropout from treatment was numerically lowest when therapy was delivered In-Home (25%) compared to Telehealth (34%) and In-Office (43%), but these differences were not statistically significant.

**Conclusions:**

CPT delivered by telehealth is an efficient and effective treatment modality for PTSD, especially considering in-person restrictions resulting from COVID-19.

**Trial registration:**

ClinicalTrials.gov ID NCT02290847 (Registered 13/08/2014; First Posted Date 14/11/2014).

**Supplementary Information:**

The online version contains supplementary material available at 10.1186/s12888-022-03699-4.

Trauma-focused psychotherapies such as cognitive processing therapy (CPT) [1] are efficacious for posttraumatic stress disorder (PTSD) in service members and veterans [2–6]. However, some service members and veterans do not avail themselves of treatment, and usage rates in practice are low [7–9]. Alarmingly, as few as 22% of soldiers who received a PTSD diagnosis only had one mental health visit [8]. Numerous barriers to therapy exist for veterans who are homebound because of injuries, illnesses, or COVID-19 precautions; who have limited transportation options; who have child or family care requirements; or who are unwilling or unable to go to U.S. Department of Veterans Affairs (VA) or Department of Defense (DoD) medical treatment facilities due to scheduling, job, or stigma concerns [10, 11]. One approach to address barriers is to deliver treatment in the patient’s home rather than in a therapist’s office [4, 5].

The COVID-19 pandemic has increased the use of telehealth to limit viral spread. Telehealth delivery of evidence-based psychotherapy from a provider’s office to a patient’s home is feasible and effective [4, 5, 12–15]. However, many previous telehealth PTSD studies used a hub-and-spoke approach, requiring veterans to travel to a local VA community-based outpatient clinic or DoD medical treatment facility, eliminating potential benefits of home-based telehealth [16, 17].

For military service members, there are many barriers (e.g., traveling to and from the clinician’s office) and potentially greater stigma (e.g., having to request supervisor or commander approval to attend a mental health appointment, being observed by fellow service members entering the mental health clinic, mental health treatment documented in the military medical record, etc.) in seeking standard in-office mental health care that may increase treatment dropout and reduce treatment effectiveness. There is evidence that home-based telehealth psychotherapy, in which patients connect with a provider via a computer-based video teleconferencing platform, is as effective as in-office care [5, 12]. Prolonged exposure, for example, was equally as effective when delivered via home-based telehealth, office-based telehealth, or in-home-in-person, among 175 veterans [5]. Delivering treatment in a patient’s home, an approach used by home healthcare workers, also increases access to care but has associated increases in provider time and cost of delivering treatment. In-home psychotherapy, in which therapists travel to patients’ homes, has proven effective for individuals with serious mental illness [18, 19] and PTSD [5]. Telehealth and in-home psychotherapy may improve treatment adherence by decreasing barriers to treatment [5, 11]. Better treatment adherence is generally associated with better treatment gains [20]. The objective of the current study was to determine if CPT delivered face-to-face in a patient’s home (In-Home CPT) or by telehealth to their home (Telehealth CPT) would result in increased acceptability, fewer dropouts, and better outcomes than routine in-office treatment (In-Office CPT).

## Methods

A detailed description of the methods of this study was previously published [21].

### Design

The study used an equipoise-stratified randomization design [22, 23] to assign participants with PTSD to receive CPT in 1 of 3 treatment modalities. Participants could agree to be randomized to any 1 of the 3 treatment modalities or opt out of 1 delivery modality and still be randomized to either of the others [22, 23]. The 3 treatment modalities included In-Office, In-Home, and Telehealth. A major strength of this design is the increased potential to improve study recruitment, because those participants who decline a treatment arm represent a proportion of participants who might not otherwise volunteer to participate in the trial [22]. Moreover, patient preferences and needs based on disability, travel limitations, or technological self-efficacy can be accommodated. It was hypothesized that (1) In-Home CPT would be more effective for the treatment of PTSD symptoms than In-Office and Telehealth CPT, (2) In-Home CPT would result in greater improvement in secondary outcomes than In-Office and Telehealth CPT, and (3) the in-home therapies (In-Home CPT and Telehealth CPT) would result in lower perceived stigma of seeking mental health care and higher treatment adherence (session attendance; out-of-session assignment completion; dose of therapy) compared to In-Office CPT.

### Participants

A description of the baseline demographics of the participants is included in Table 1. Participant recruitment occurred between September 19, 2014, and June 29, 2018, with final follow-up assessment on June 30, 2019. Participants were active duty U.S. military members (*n* = 20) and veterans (*n* = 100) who were 18 years of age or older seeking treatment for PTSD. Eligibility required experience of a *Diagnostic and Statistical Manual of Mental Disorders* (5th ed.; *DSM-5*) Criterion A traumatic event during a military deployment. Participation was initially limited to post-9/11/2001 veterans, but this was later expanded to include veterans of all war eras to increase recruitment. The diagnosis of PTSD, however, could have been based on a different Criterion A event (e.g., childhood abuse). Other inclusion criteria were stability on psychotropic medications and living within a 60-mile radius of the university offices, the established maximum traveling distance for the In-Home CPT arm. Exclusion criteria were as follows: suicide or homicide risk warranting crisis intervention; endorsing items pertaining to danger of violence (e.g., unsafe neighborhood, aggressive dogs, etc.) that might place study therapists at personal risk if providing in-person treatment in the patient’s home; significant alcohol and/or substance use that might interfere with practice assignments and therapy attendance; active psychosis; and significantly impaired cognitive functioning.

**Table 1:** Baseline Demographic Characteristics of Participants by Treatment Randomization Strata

N	Total Sample		In-Home Strata		In-Office Strata		Telehealth Strata				
	120		32		44		44				
	Mean	SD	Mean	SD	Mean	SD	Mean	SD	F-ratio	df	p
**Age**	40.5	10.5	41.9	10.9	38.5	11.8	41.4	8.6	1.26	2, 117	0.289
**Years of Service**	14.0	8.0	15.6	7.8	11.1	7.1	15.7	8.3	4.81	2, 117	0.010
**Outcome Measures**											
CAPS-5	37.8	9.0	37.6	9.6	35.6	7.8	37.3	9.9	0.56	2, 117	0.575
PCL-5	49.9	13.4	52.0	12.7	48.5	12.8	49.7	14.5	0.63	2, 117	0.532
BDI-II	33.8	11.0	32.4	12.9	35.2	8.6	33.5	11.8	0.58	2, 117	0.559
	N	%	N	%	N	%	N	%	χ2		p
**Male**	106	88%	28	88%	42	95%	36	82%	4.00	2	0.135
**Married**	93	78%	26	81%	33	75%	34	77%	0.42	2	0.812
**Education**									8.09	8	0.425
High School	8	7%	2	6%	2	5%	4	9%			
Some College	40	33%	9	28%	20	45%	11	25%			
Associates	25	21%	8	25%	9	20%	8	18%			
4-year College	33	28%	10	31%	7	16%	16	36%			
Postgraduate	14	12%	3	9%	6	14%	5	11%			
**Ethnicity/Race**									9.21	6	0.162
Black	20	17%	8	25%	5	11%	7	16%			
Hispanic	50	42%	12	38%	20	45%	18	41%			
White	44	37%	11	34%	14	32%	19	43%			
Other	6	5%	1	3%	5	11%	0	0%			
**Times Deployed**									14.94	8	0.060
0	13	11%	3	9%	6	14%	4	9%			
1	41	34%	6	19%	17	39%	18	41%			
2	32	27%	13	41%	7	16%	12	27%			
3	20	17%	9	28%	6	14%	5	11%			
4+	14	12%	1	3%	8	18%	5	11%			
**Service Branch**									2.23	6	0.897
Air Force	19	16%	5	16%	5	11%	9	20%			
Army	67	56%	17	53%	25	57%	25	57%			
Marines	18	15%	5	16%	7	16%	6	14%			
Navy	16	13%	5	16%	7	16%	4	9%			
**Military Grade**									10.04	6	0.123
E-1 to -E-3	2	2%	0	0%	2	5%	0	0%			
E-4 to E-6	76	63%	20	63%	31	70%	25	57%			
E-7 to E-9	30	25%	11	34%	6	14%	13	30%			
Officer	12	10%	1	3%	5	11%	6	14%			
**Duty**									1.47	4	0.831
Combat Arms	37	31%	11	34%	12	27%	14	32%			
Combat Support	36	30%	9	28%	12	27%	15	34%			
Service Support	47	39%	12	38%	20	45%	15	34%			

Abbreviations: *BDI-II* Beck Depression Inventory, Second Edition; *CAPS-5* Clinician-Administered PTSD Scale for *DSM-5*; *E-1 to -E-3* junior enlisted military; *E-4 to E-6* junior noncommissioned officers; *E-7 to E-9 m* senior noncommissioned officers; *PCL-5* PTSD Checklist for *DSM-5*

This study was approved by the Institutional Review Boards at the University of Texas Health Science Center at San Antonio, Duke Health, and the Boston VA. The U.S. Army Medical Research and Materiel Command (now the U.S. Army Medical Research and Development Command) Human Research Protection Office reviewed the regulatory determinations. Adverse events (AEs) were monitored during each participant contact using an AE monitoring program used in previous clinical trials [24]. All subjects provided written informed consent after receiving a complete description of the study.

### Measures

The primary outcome measures included the PTSD Checklist for DSM-5 (PCL-5) [25] to assess self-reported changes in PTSD symptom severity at 13 time points from baseline to the 6-month follow-up, and the Clinician-Administered PTSD Scale for DSM-5 (CAPS-5) [26] to assess clinician-interviewed PTSD symptom severity and diagnosis at 4 time points from baseline to 1-, 3-, and 6-month follow-up. All clinical evaluators completed extensive training, certification, and repeated calibration training to ensure the fidelity of the assessments [27] and were blinded to participant treatment condition. The Beck Depression Inventory II (BDI-II) [28] was used to assess self-reported symptoms of depression.

### Procedures

**Fig. 1:**
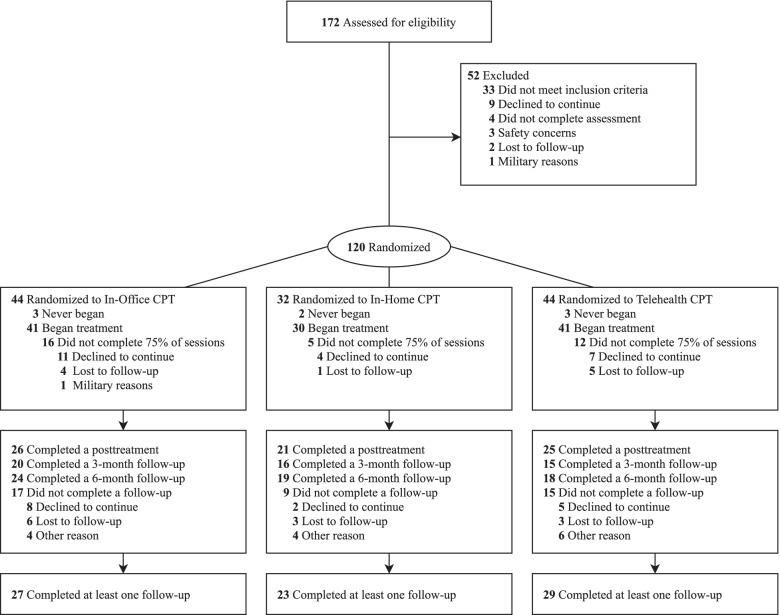
CONSORT chart. CPT = Cognitive Processing Therapy

Participants were recruited (*N* = 120) from advertisements and referrals from VA and DoD providers (see Fig. 1, CONSORT flow chart of the study). All participants met criteria for PTSD established during the baseline assessment by independent evaluators. If eligible, participants were given the choice to be randomized to 1 of the 3 treatment arms using 1:1:1 randomization or to opt out of 1 treatment arm and be randomized 1:1 to either of the other 2.

### Treatment

All participants received CPT [1] delivered in 12 sessions, 60 min each, delivered twice a week for 6 weeks. Four therapist treated participants in all 3 arms of the study and attended weekly consultation calls led by CPT experts. Therapy sessions were audio recorded, and a random sample of 5% of all sessions (55 one-hour sessions) were rated for protocol adherence and therapist competence by CPT. Adherence was excellent, with 98.9% of CPT elements delivered and no proscribed elements performed. Competence was rated on a 5-point Likert scale (1 = “Poor” through 5 = “Excellent”). The ratings distribution was negatively skewed, with 86.4% of CPT elements (M = 4.43 ± 0.51) and 87.9% of non-specific essential elements (M = 4.61 ± 0.52) rated between Good (“4”) and Excellent (“5”). Inter-rater reliability was good (κ = .88) between 2 raters.

In-Office CPT was conducted in typical face-to-face fashion in university offices. In-Home CPT was conducted by therapists traveling to participants’ homes during regular business hours. Prior to the first In-Home CPT session, home safety precautions for therapist visits were discussed among the study team members, and confidentiality and privacy issues were discussed with the patient. Telehealth CPT was conducted through a computer-based video link connection from the therapist’s office to the participant’s home. Participants who did not have adequate computer resources were loaned telehealth equipment.

### Statistical analysis

Statistical analysis of data collected in an equipoise-stratified randomization design is quite complicated [22]. Intent-to-treat (ITT) analyses were used and included all data without regard to engagement in treatment or study participation. The equipoise randomization design allowed participants to opt out of any 1 of the 3 treatment formats, resulting in the 4 *equipoise strata* summarized in Table 2. The proportions opting out of each arm were compared (1-way chi-square tests). Attrition was analyzed using survival analysis of sessions to all-cause discontinuation (see Supplemental Fig. 1), and a contingency table analyzed dropout operationalized as attending fewer than 75% (9/12) of the sessions. Baseline demographics, military service history, and baseline symptom severity were examined as predictors of dropout with contingency table and logistic regression analyses.

**Table 2:** Treatment Strata, Sample Sizes, Treatment Arms Declined, and Dropped Out from Treatment

Stratum	Sample Size				Treatment Arm Declined		Dropped Out of Treatment	
	In-Home	In-Office	Tele-health	Total	N	%	N	%
A: All Formats*	17	17	17	51	N/A	N/A	N/A	N/A
B: No Telehealth	5	7	–	12	12/69	17%	15/44	34%
C: No In-Office	10	–	10	20	20/69	29%	19/44	43%
D: No In-Home	–	20	17	37	37/69	54%	8/32	25%
Totals	32	44	44	120	69/120	57%	42/120	35%

*Note: All Formats includes those participants willing to be randomized to any of the three treatment formats (In-Home, In-Office, or Telehealth)

Outcome analyses addressed changes in symptoms of PTSD (PCL-5, CAPS-5) and depression (BDI-II). Total scores on the self-report PCL-5 were the primary outcome measure of PTSD symptoms. PCL-5 assessments were completed more frequently (13 times: baseline, weekly during treatment, and monthly for 6 months after treatment) than the CAPS-5 (4 times), and thus provided a more complete picture of trajectories over time with greater statistical power. The BDI-II was completed weekly during treatment and 3 times posttreatment.

Symptom outcomes were analyzed in 2 ways. One analysis used data from all participants (full sample) without regard for randomization. Referring to Table 2, for example, the full-sample analyses of the In-Home arm used data from strata A, B, and C. A planned contrast was used for an omnibus test of the differences in slopes among the 3 treatment arms with follow-up pairwise contrasts by t test. The full-sample analyses included separate intercepts for the 3 treatment arms because the samples were not fully randomized.

The full-sample analyses have the most precision and broadest generalizability. However, they may not be a valid basis for comparisons of treatments because when the opt-out strata are ignored, the analysis is based on partially non-randomized samples. To ensure that differences between treatments are not confounded with differences in the patient samples, the comparisons of any two treatments needed to be based on fully randomized samples. The fully randomized equipoise-stratified comparison between In-Home and In-Office treatment, for example, would use only data from strata A and B, in this instance a reduction in sample size from 76 to 46. The equipoise-stratified analyses are more rigorous for comparing treatment arms than the full-sample analyses because they are based on fully randomized samples, but they obviously sacrifice both generalizability and statistical power. The equipoise-stratified comparison analyses assumed no baseline differences between treatment arms because the samples were fully randomized and baseline differences could only be chance fluctuations.

Two secondary outcomes were the Reliable Change Index (RCI) [29], on the PCL-5 and diagnostic remission on the CAPS-5. Based on data from this trial, an improvement of 10 points or more in PCL-5 total score was expected to occur by chance no more than 5% of the time solely due to measurement error. Given as many as 6 assessments during treatment, the RCI was defined as an improvement of 10 or more points that was sustained at all subsequent assessments, so transient improvement with subsequent worsening did not qualify. Remission was defined as loss of PTSD diagnosis on the CAPS-5 at the 1-month posttreatment assessment. Contingency table analysis was used to examine RCI and diagnostic remission. Statistical significance for hypothesis tests was set at unadjusted *p* = .05. All analyses were done using software from the SAS 9.4 statistical library (SAS Institute).

## Results

### Final randomization allocation

More than half of the participants (*n* = 69/120; 57%) opted out of 1 of the treatment arms, which resulted in a final randomization of fewer participants into the In-Home arm (*n* = 32) compared to the In-Office (*n* = 44) and Telehealth (*n* = 44) arms. This occurred because In-Home treatment was the modality most often declined by participants (see Table 2 and Fig. 1).

The acceptability of the 3 treatment options differed significantly (χ2 = 14.2, df = 2, *p* = 0.0008). Among those opting out of 1 delivery modality, most refused In-Home treatment (37/69; 54%) followed by In-Office (20/69; 29%) and Telehealth (12/69; 17%). The most common reasons given for opting out of In-Home treatment were the presence of “distractions” at home such as children, spouses, other adults, and pets, as well as the perceived stigma of receiving mental health treatment in their home. The most common explanation for opting out of In-Office treatment was inconvenience, typically regarding difficulties of transportation. No In-Office participants refused treatment because of concerns of stigma. The most common reason for opting out of telehealth was because of perceived impersonality. No participants refused telehealth for privacy or personal security concerns.

### Attrition

The observed at each data point are included in Supplemental Table 1. The Kaplan-Meier survival functions for all-cause discontinuation are displayed in Supplemental Fig. 1 based on a product-limit survival analysis of number of sessions attended. A total of 42 patients (35%) dropped out of treatment, including 11 who never began. The proportions dropping out were lowest for In-Home (8/32; 25%), intermediate for Telehealth (15/44; 34%), and greatest for In-Office (19/44; 43%). However, these differences were not statistically significant (log-rank χ2 = 2.69, df = 2, *p* = .26). Frequency analysis of the proportions completing 9 or more sessions in the 3 treatment arms yielded similar results (χ2 = 3.74, df = 2, *p* = .175). None of the demographics (age, education, ethnicity/race, sex, marital status), military characteristics (years of service, military pay grade, number of deployments, military occupation), or baseline clinical measures were significant predictors of discontinuation by univariate log-rank test (all *p* > .05), and none of the predictors remained in proportional hazard regression models using either forward or backward selection.

### PTSD symptom improvement

On the PCL-5, improvement was statistically large in all 3 treatment arms (all ps < .0001). Table 3 and Supplemental Fig. 2 include the results for the PCL-5 in the full sample PCL-5 from baseline to posttreatment and from posttreatment to 6-months follow-up. Improvement on the PCL-5 was about twice as large in the In-Home (*d* = 2.1) and Telehealth (d = 2.0) formats compared to In-Office (*d* = 1.3). Both of those differences between treatments were statistically large (*d* = .8 and .7) and significant (*p* = .009 and .014). The difference between In-Home and Telehealth PCL-5 outcomes was negligible (*p* = 0.77, *d* = −.08). The differences between treatment arms on the PCL-5 dissipated by the 6-month follow-up point.

**Table 3:** PTSD Checklist for *DSM-5* (PCL-5) Change from Baseline to Posttreatment and from Posttreatment to 6-Months Follow-Up with Pairwise Differences for the Full Sample

Treatment Arm	Baseline to Posttreatment			Posttreatment to 6-month		
	Estimate (StdErr)	t	Cohen’s d (95% CL)	Estimate (StdErr)	t	Cohen’s d (95% CL)
Telehealth	−26.6 (2.56)	−10.41**	−2.0 (− 2.4/− 1.6)	4.1 (3.03)	1.36	0.31 (− 0.1/+ 0.8)
In-Home	− 27.8 (2.81)	−9.86**	−2.1 (− 2.5/− 1.7)	3.5 (3.19)	1.10	0.26 (− 0.2/+ 0.7)
Office	−17.6 (2.64)	−6.69	− 1.3 (− 1.7/− 0.9)	−1.9 (2.84)	−0.66	− 0.14 (− 0.6/+ 0.3)
Pairwise Differences						
In-Home v. Office	−10.1 (3.86)	− 2.63*	−0.76 (− 1.3/− 0.2)	5.4 (4.27)	1.26	0.40 (− 0.2/+ 1.0)
Telehealth v. Office	−9.0 (3.67)	−2.45*	− 0.67 (− 1.2/− 0.1)	6.0 (4.15)	1.44	0.45 (− 0.2/+ 1.1)
In-Home v Telehealth	− 1.1 (3.80)	−0.29	− 0.08 (− 0.6/+ 0.5)	−0.6 (4.39)	− 0.14	−0.05 (− 0.7/+ 0.6)

Note: Estimates from piecewise linear model. Omnibus test of differences among treatment arms: baseline to post (F = 4.34, df = 2, 812, *p* = .013) and post to 6-month follow-up (F = 1.27, df = 2, 812, *p* = .282). Cohen’s d is standardized by the baseline PCL-5 standard deviation 13.35. All t-test df = 812.*p < .05, **p < .001

Supplemental Table 2 presents the PCL-5 results from those who opted out of one treatment. The only notable change from the full-sample analyses is that the *p* value for the difference between Telehealth and In-Office increased from .015 to .075. As in the full-sample analyses, improvements were large and significant in all 3 treatment arms, and In-Home and Telehealth arms did not differ.

Analysis of the PCL-5 using the RCI showed the proportions achieving reliable change at posttreatment were 78% (25/32) for In-Home, 59% (26/44) for Telehealth, and 48% (21/44) for In-Office and these differences were statistically significant (χ2 = 7.16, df = 2, *p* = 0.028). The pairwise equipoise-stratified analyses confirmed that the proportion achieving sustained RCI with In-Home treatment was significantly larger than In-Office (χ2 = 6.92, df = 1, *p* = .009). Telehealth was intermediate and did not differ significantly from either In-Home (χ2 = 3.20, df = 1, *p* = .074) or In-Office (χ2 = 1.72, df = 1, *p* = .19).

A similar pattern of outcomes was seen on the CAPS-5 (see Table 4, Supplemental Table 3, and Supplemental Fig. 3). Pre-post improvement in the CAPS-5 total was significant in all 3 treatment arms (Telehealth 13.4 ± 2.8, In-Home, 15.4 ± 3.1, In-Office 10.4 ± 2.8, all *p* < .0005). Improvement was numerically largest with In-Home treatment and smallest with In-Office, but differences between treatment arms were not significant (all *p* > .20). At posttreatment, the proportions no longer meeting CAPS-5 diagnostic criteria for PTSD were 62% for In-Home, 58% for In-Office, and 48% for Telehealth, a nonsignificant difference (χ2 = 0.97, df = 2, *p* = .62).

**Table 4:** Clinician-Administered PTSD Scale for *DSM-5* (CAPS-5) Change from Baseline to Posttreatment and from Posttreatment to 6-Months Follow-Up with Pairwise Differences for the Full Sample

Treatment Arm	Baseline to Posttreatment			Posttreatment to 6-month		
	Estimate (StdErr)	t	Cohen’s d (95% CL)	Estimate (StdErr)	t	Cohen’s d (95% CL)
Telehealth	−13.4 (2.78)	−4.83**	−1.5 (− 2.1/− 0.9)	−2.5 (1.99)	−1.26	−0.3 (− 0.7/+ 0.2)
In-Home	− 15.4 (3.06)	−5.04**	−1.7 (− 2.4/− 1.0)	−1.1 (1.90)	−0.60	− 0.1 (− 0.5/+ 0.3)
In-Office	−10.4 (2.80)	− 3.74**	− 1.2 (− 1.2/− 0.5)	−2.0 (1.67)	−1.21	− 0.2 (− 0.6/+ 0.1)
Pairwise Differences						
In-Home v. In-Office	−5.0 (4.15)	− 1.20	− 0.6 (− 1.5/+ 0.4)	0.9 (2.53)	0.35	0.1 (−0.5/+ 0.7)
Telehealth v.In- Office	−3.0 (3.94)	−0.75	−0.3 (− 1.2/+ 0.5)	−0.5 (2.60)	− 0.19	−0.1 (− 0.6/+ 0.5)
In-Home v Telehealth	−2.0 (4.14)	− 0.48	−0.2 (− 1.1/+ 0.7)	1.4 (2.75)	−0.50	0.2 (− 0.5/+ 0.8)

Note: Estimates from piecewise linear model. Omnibus test of differences among treatment arms: baseline to post (F = 0.74, df = 2, 117, *p* = 0.48) and post to 6-month follow-up (F = 0.13, df = 2, 117, *p* = .88.). Cohen’s d is standardized by the baseline CAPS-5 standard deviation 9.01. All t-test df = 117. *p < .05, **p < .001

### Depression symptom improvement

Supplemental Table 4 and Supplemental Fig. 4 present the estimated least-square means on the BDI-II over time. Similar to PTSD outcomes, improvement in depression was significant in all 3 treatment arms, but it was considerably larger in the In-Home (d = 1.2) and Telehealth (d = 1.1) arms than In-Office treatment (d = .52). The differences between In-Office and the other 2 formats were both statistically large (Cohen’s d = .7), and they both remained significant (*p* < .01) in the pairwise equipoise stratified analyses despite the reduction in sample sizes.

### Therapist and patient time commitments by treatment arm

An anecdotal finding was that In-Office and In-Home treatment delivery required about twice as much time commitment (i.e., 2 h per treatment session) for the therapist or patient compared to the Telehealth modality (1 h per session). It was estimated that there was an average of 1 h of commuting time (30 min to and from the treatment location) for the patient to travel to the therapist’s office (In-Office CPT) or the therapist to travel to the patient’s home (In-Home CPT). As a result, the Telehealth CPT modality required a total time commitment of 12 h for both the therapist and patient compared to 24 h for the patient or therapist for the other modalities.

### Adverse events

Adverse events (AEs) were assessed by the therapist once per week during the intervention period. During the treatment phase, 53% (63/120) of participants reported a total of 133 AEs. Most AEs were general medical or health conditions that were judged to be “unrelated” to the study procedures; however, 28% (33/120) of participants reported a total of 51 AEs that were at least “possibly” related. The most common related AEs reported by more than 3 participants were nightmares (7.5%), sleep difficulty (5.8%), depression (5.0%), anxiety (4.2%), and irritability (4.2%). None of these AEs differed significantly by group after adjustment for the numbers of participants in each group.

## Discussion

This randomized clinical trial was the first to use an equipoise-stratified randomization design to evaluate efficacy, acceptability, and dropout of CPT delivered via 3 different treatment modalities. The results indicated that the majority of patients had clinically significant improvements in PTSD symptom severity on the PCL-5 as measured by the RCI [29], including 78% with In-Home CPT, 59% with Telehealth CPT, and 48% with In-Office CPT. At the posttreatment follow-up point, improvement on the PCL-5 was about twice as large in the In-Home and Telehealth formats compared to In-Office. However, these same differences were not found on the CAPS-5 at posttreatment, and there were no differences between the treatment modalities on the PCL-5 or CAPS-5 at the 6-month follow-up point. The randomization scheme resulted in fewer participants being assigned to the In-Home CPT arm (*n* = 32 versus *n* = 44 for both of the other two arms) because it was the treatment modality that was least acceptable and most often declined by participants.

There were several unexpected findings in the In-Home arm of the study. In-Home CPT was the treatment modality hypothesized to offer the greatest potential to overcome common barriers to PTSD treatment, such as accessibility or stigma. However, out of the 57% (69/120) of participants who declined a treatment arm, In-Home CPT was declined almost twice as often (54%) as In-Office CPT (29%) and more than 3 times as often as Telehealth CPT (17%). This unexpected finding suggests that patient-identified in-home distractions or the stigma of having a mental health provider come to their home may have been greater than anticipated. Other unidentified factors such as privacy concerns and fear of being overheard may have also negatively affected patient willingness to receive care within the home. Paradoxically, although the In-Home CPT modality was most likely to be declined, it also was the modality with the greatest proportion of individuals achieving reliable change in self-reported PTSD symptoms, from baseline to posttreatment. The dropout rate with In-Home CPT also appeared to be lower (25%; 8/32) than with Telehealth (34%; 15/44) and In-Office (43%; 19/44), but these differences were statistically nonsignificant. While patients willing to receive PTSD treatment in their home may be more likely to receive a full dose of therapy and experience greater symptom reductions, we do not know if the lower rates of accepting In-Home care may have produced a biased sample of more motivated patients who responded better.

Another unexpected finding was that the In-Home treatment modality may expose therapists to more personal risks than are likely to occur with the Telehealth or In-Office formats. Although considerable safeguards were implemented to help ensure that patients’ homes, neighborhoods, and the overall environment were safe for therapists, 1 traumatic event occurred during an In-Home treatment session (suicide of a family member; determined to not to be related to the treatment of the patient) that was distressing for the therapist. It is important that clinics employing in-home treatments develop safety protocols and be aware that, despite all planning, some events cannot be prevented.

The high dropout in the In-Office arm (43%) is consistent with other reports, which have highlighted the high dropout rates found in standard, in-office, trauma-focused treatments for PTSD [8]. However, the findings from the current study suggest that the high dropout rates with many trauma-focused PTSD treatments may be more related to the treatment delivery modality than to factors related to the treatment itself. Moreover, dropout rates are not necessarily indicative of treatment ineffectiveness. As many as 33–55% of civilians who dropped out of a CPT clinical trial demonstrated clinically significant reductions in symptoms, or met good end-state functioning related to depression or PTSD [30]. A recent study of variable-length CPT found 28% of dropouts were found to have remitted from their PTSD and 23% made clinically significant improvements [31].

The study had several limitations. Although participants in all treatment arms maintained significant reductions on the PCL-5 through the end of the 6-month follow-up period, the significant differences between treatment arms dissipated. Regarding the research design, a standard 3-armed randomized clinical trial may have been a more scientifically rigorous design, and the data analyses would have been more straightforward. However, as proposed by Lavori et al. [22], a strength of the equipoise-stratified randomization design was that it allowed for 57% of the participants in the current study to decline 1 treatment arm. This suggests that the majority of the patients may have declined to volunteer to participate in the study had this been a standard 3-arm randomized clinical trial. That could have led to twice as many potential participants needing to be screened to reach the same sample size.

Another limitation of the study was the lack of specific data on patient treatment preferences. Although data were collected on why participants declined participation in specific treatment arms, data were not gathered on which treatment arm participants might have most preferred. Additionally, there are practical limitations associated with the delivery of evidence-based therapies for PTSD in each of the treatment arms. The additional travel time required for therapists and patients for the In-Home and In-Office treatments is a limitation of these modalities, and reimbursement for travel expenses may not always be available. Regarding telehealth treatment, some patients may not have computer equipment, tablets, or broadband Internet, and lending equipment may not be possible for certain clinics. However, since the onset of COVID-19 restrictions in the spring of 2020, the use of video communications for personal and professional purposes is thought to have increased exponentially for both therapists and patients.

Although the strongest PTSD outcomes and lowest dropout rates were found using the In-Home CPT delivery format, it was the least acceptable treatment to patients. It also required double the amount of therapist time, had unexpected distractions and stigmas associated with it for the patients, and may be associated with an increased risk of exposure by the therapists to unexpected events in the patient’s home. As a result, the in-home treatment modality should probably be limited to patients who are homebound or have other extreme travel limitations. Future studies should examine the potential for improved treatment outcomes when modalities are blended to accommodate patients. For example, patients’ scheduling conflicts may require clinicians to switch from in-office to telehealth, which was common during the COVID-19 pandemic.

In terms of PTSD symptom improvement, treatment acceptability, treatment retention, and overall estimated cost in dollars and time commitment, the results provide strong support for the use of telehealth for the treatment of PTSD. This is particularly relevant with the recent worldwide COVID-19 pandemic that has forced many behavioral health providers to switch to telehealth for much of their work.

## Supplementary Information

**Additional file 1: Supplementary Figure 1.** All-cause discontinuation from treatment for the full sample.

**Additional file 2: Supplementary Figure 2.** Changes in PCL-5 totals from baseline to 6 months posttreatment for the full sample. BL = baseline; M = month; PCL-5 = PTSD Checklist for *DSM-5*; PTX = posttreatment; S = session.

**Additional file 3: Supplementary Figure 3.** Changes in CAPS-5 totals from baseline to 6 months posttreatment for the full sample. CAPS-5 = Clinician-Administered PTSD Scale for *DSM-5.*

**Additional file 4: Supplementary Figure 4.** Changes in BDI-II totals from baseline to 6 months posttreatment for the full sample. BDI-II = Beck Depression Index II; BL = baseline; M = month; PTX = posttreatment; S = session.

**Additional file 5: Supplementary Table 1.** PTSD Checklist for DSM-5 (PCL-5) change during treatment with pairwise differences for full sample as compared with equipoise-stratified samples.

**Additional file 6: Supplementary Table 2.** Change in Clinician-Administered PTSD Scale for DSM-5 (CAPS-5) from baseline to posttreatment for full-sample compared with equipoise-stratified samples.

**Additional file 7: Supplementary Table 3.** Change in Beck Depression Inventory, Second Edition (BDI-II) from baseline to posttreatment for full-sample as compared with equipoise-stratified samples.

**Additional file 8: Supplemental Table 4.** Number of participants who completed assessment visit during study.

## Data Availability

Data are available upon request from Repository@strongstar.org.

## Abbreviations

AE: Adverse event
BDI-II: Beck Depression Inventory, Second Edition
CAPS-5: Clinician-Administered PTSD Scale for *DSM-5*
COVID-19: Coronavirus disease
CPT: Cognitive processing therapy
E-1 to -E-3: Junior enlisted military
E-4 to E-6: Junior noncommissioned officers
E-7 to E-9: Senior noncommissioned officers
ITT: Intent-to-treat
M: Mean
PCL-5: PTSD Checklist for *DSM-5*
PTSD: Posttraumatic stress disorder
RCI: Reliable Change Index
VA: U.S. Department of Veterans Affairs

## References

[1] Resick PA, Monson CM, Chard KM (2017). Cognitive processing therapy for PTSD: a comprehensive manual.

[2] Kaysen D, Schumm J, Pedersen ER (2014). Cognitive processing therapy for veterans with comorbid PTSD and alcohol use disorders. Addict Behav.

[3] Monson CM, Schnurr PP, Resick PA (2006). Cognitive processing therapy for veterans with military-related posttraumatic stress disorder. J Consult Clin Psychol.

[4] Morland LA, Mackintosh MA, Greene CJ (2014). Cognitive processing therapy for posttraumatic stress disorder delivered to rural veterans via telemental health: a randomized noninferiority clinical trial. J Clin Psychiatry.

[5] Morland LA, Mackintosh MA, Glassman LH (2020). Home-based delivery of variable length prolonged exposure therapy: a comparison of clinical efficacy between service modalities. Depress Anxiety.

[6] Resick PA, Wachen JS, Mintz J (2015). On behalf of the STRONG STAR consortium: a randomized clinical trial of group cognitive processing therapy compared with group present-centered therapy for PTSD among active duty military personnel. J Consult Clin Psychol.

[7] Resick PA, Wachen JS, Dondanville KA (2017). And the STRONG STAR consortium: effect of group vs individual cognitive processing therapy in active-duty military seeking treatment for posttraumatic stress disorder: a randomized clinical trial. JAMA. Psychiatry.

[8] Hoge CW, Grossman SH, Auchterlonie JL (2014). PTSD treatment for soldiers after combat deployment: low utilization of mental health care and reasons for dropout. Psychiatr Serv.

[9] Peterson AL, Luethcke CA, Borah EV (2011). Assessment and treatment of combat-related PTSD in returning war veterans. J Clin Psychol Med Settings.

[10] Tanielian TL, Jaycox L (2008). RAND Corporation: invisible wounds of war: psychological and cognitive injuries, their consequences, and services to assist recovery.

[11] Greene-Shortridge TM, Britt TW, Castro CA (2007). The stigma of mental health problems in the military. Mil Med.

[12] Acierno R, Gros DF, Ruggiero KJ (2016). Behavioral activation and therapeutic exposure for posttraumatic stress disorder: a noninferiority trial of treatment delivered in person versus home-based telehealth. Depress Anxiety.

[13] Germain V, Marchand A, Bouchard S (2009). Effectiveness of cognitive behavioural therapy administered by videoconference for posttraumatic stress disorder. Cogn Behav Ther.

[14] Hassija C, Gray MJ (2011). The effectiveness and feasibility of videoconferencing technology to provide evidence-based treatment to rural domestic violence and sexual assault populations. Telemed J E Health.

[15] Morland LA, Mackintosh MA, Rosen CS (2015). Telemedicine versus in-person delivery of cognitive processing therapy for women with posttraumatic stress disorder: a randomized noninferiority trial. Depress Anxiety.

[16] Tuerk PW, Yoder M, Ruggiero KJ (2010). A pilot study of prolonged exposure therapy for posttraumatic stress disorder delivered via telehealth technology. J Trauma Stress.

[17] Morland LA, Hynes AK, Mackintosh MA (2011). Group cognitive processing therapy delivered to veterans via telehealth: a pilot cohort. J Trauma Stress.

[18] Velligan DI, Diamond PM, Maples NJ (2008). Comparing the efficacy of interventions that use environmental supports to improve outcomes in patients with schizophrenia. Schizophr Res.

[19] Velligan DI, Diamond P, Mueller J (2009). The short-term impact of generic versus individualized environmental supports on functional outcomes and target behaviors in schizophrenia. Psychiatry Res.

[20] Berke DS, Kline NK, Wachen JS (2019). For the STRONG STAR consortium. Predictors of attendance and dropout in three randomized controlled trials of PTSD treatment for active duty service members. Behav Res Ther.

[21] Peterson AL, Resick PA, Mintz J (2018). For the STRONG STAR consortium: design of a clinical effectiveness trial of in-home cognitive processing therapy for combat-related PTSD. Contemp Clin Trials.

[22] Lavori PW, Rush AJ, Wisniewski SR (2001). Strengthening clinical effectiveness trials: equipoise-stratified randomization. Biol Psychiatry.

[23] Shalev AY, Ankri Y, Israeli-Shalev Y (2012). Prevention of posttraumatic stress disorder by early treatment: results from the Jerusalem trauma outreach and prevention study. Arch Gen Psychiatry.

[24] Peterson AL, Roache JD, Raj J, Young-McCaughan S (2013). For the STRONG STAR consortium. The need for expanded monitoring of adverse events in behavioral health clinical trials. Contemp Clin Trials.

[25] Weathers FW, Litz BT, Keane TM (2013). The PTSD checklist for DSM-5 (PCL-5).

[26] Weathers FW, Blake DD, Schnurr PP (2013). The clinician administered PTSD scale for DSM-5 (CAPS-5).

[27] Barnes BJ, Presseau C, Jordan AH (2019). And the consortium to alleviate PTSD: common data elements in the assessment of military-related PTSD research applied in the consortium to alleviate PTSD. Mil Med.

[28] Beck AT, Steer RA, Brown GK (1996). Manual for the BDI-II.

[29] Jacobson NS, Truax P (1991). Clinical significance: a statistical approach to defining meaningful change in psychotherapy research. J Consult Clin Psychol.

[30] Szafranski DD, Smith BN, Gros DF, Resick PA (2017). High rates of PTSD treatment dropout: a possible red herring?. J Anxiety Disord.

[31] Resick PA, Wachen JS, Dondanville KA (2021). For the STRONG STAR Consortium. Variable-length cognitive processing therapy for posttraumatic stress disorder in active duty military: outcomes and predictors. Behav Res Ther.

