# Supplementary figures and images for: In-office, in-home, and telehealth cognitive processing therapy for posttraumatic stress disorder in veterans: a randomized clinical trial
Source: BMC Psychiatry. 2022 Jan 17;22:41. doi: 10.1186/s12888-022-03699-4 (PMC8763446; doi:10.1186/s12888-022-03699-4)

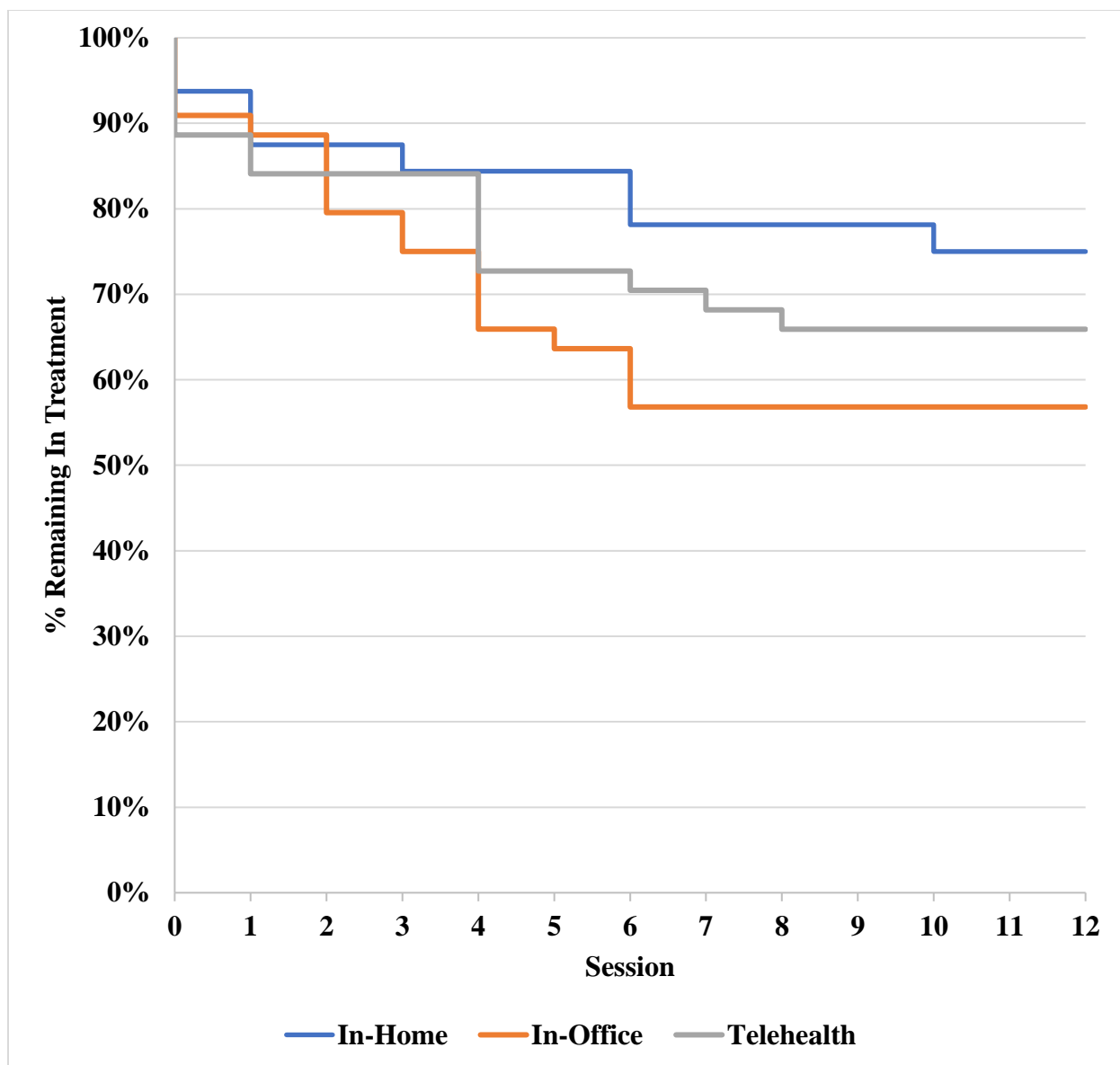

Supplement: Supplementary file 1 — Additional file 1: Supplementary Figure 1. All-cause discontinuation from treatment for the full sample. [file 12888_2022_3699_MOESM1_ESM.pdf]

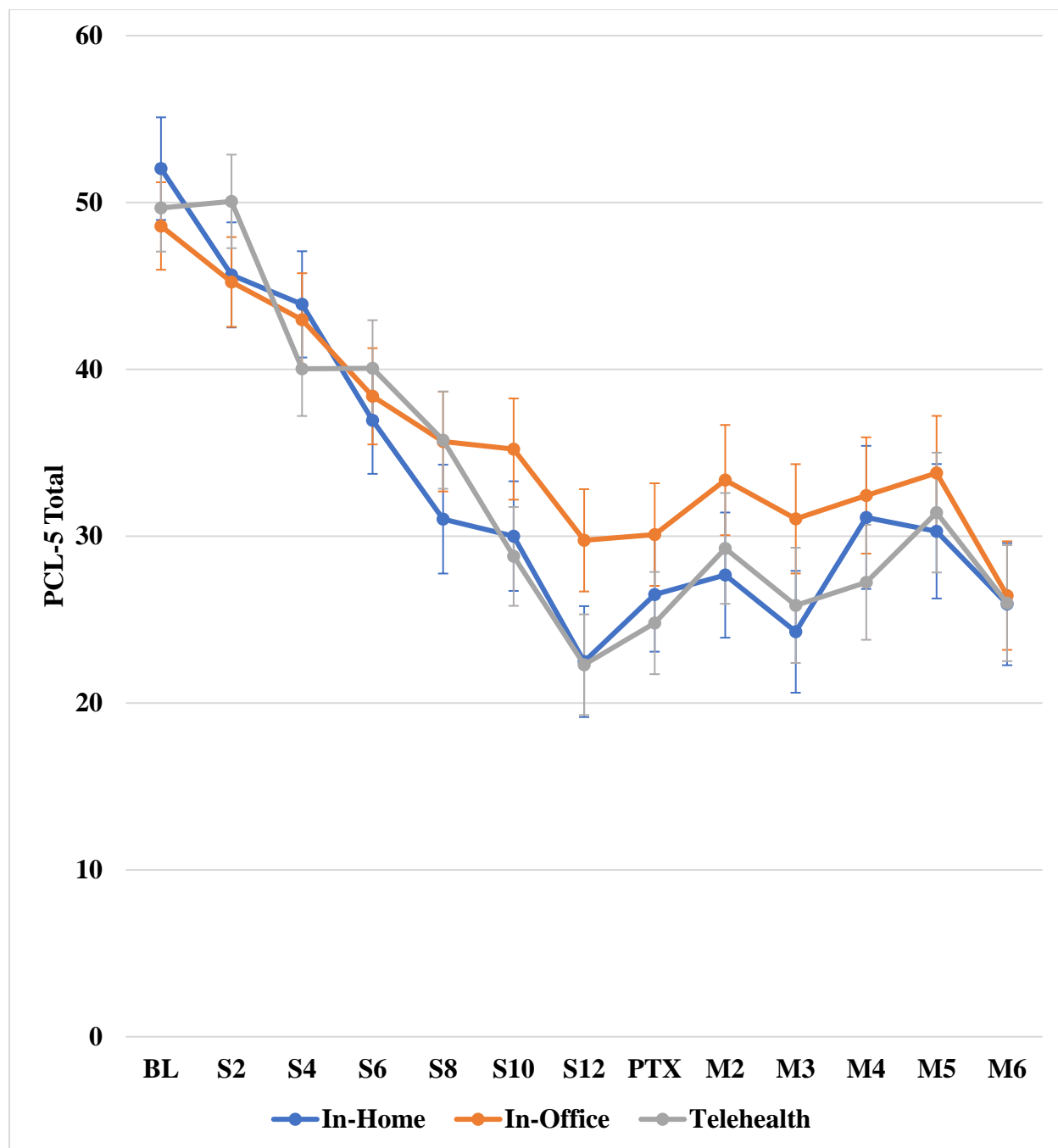

Supplement: Supplementary file 2 — Additional file 2: Supplementary Figure 2. Changes in PCL-5 totals from baseline to 6 months posttreatment for the full sample. BL = baseline; M = month; PCL-5 = PTSD Checklist for DSM-5; PTX = posttreatment; S = session. [file 12888_2022_3699_MOESM2_ESM.pdf]

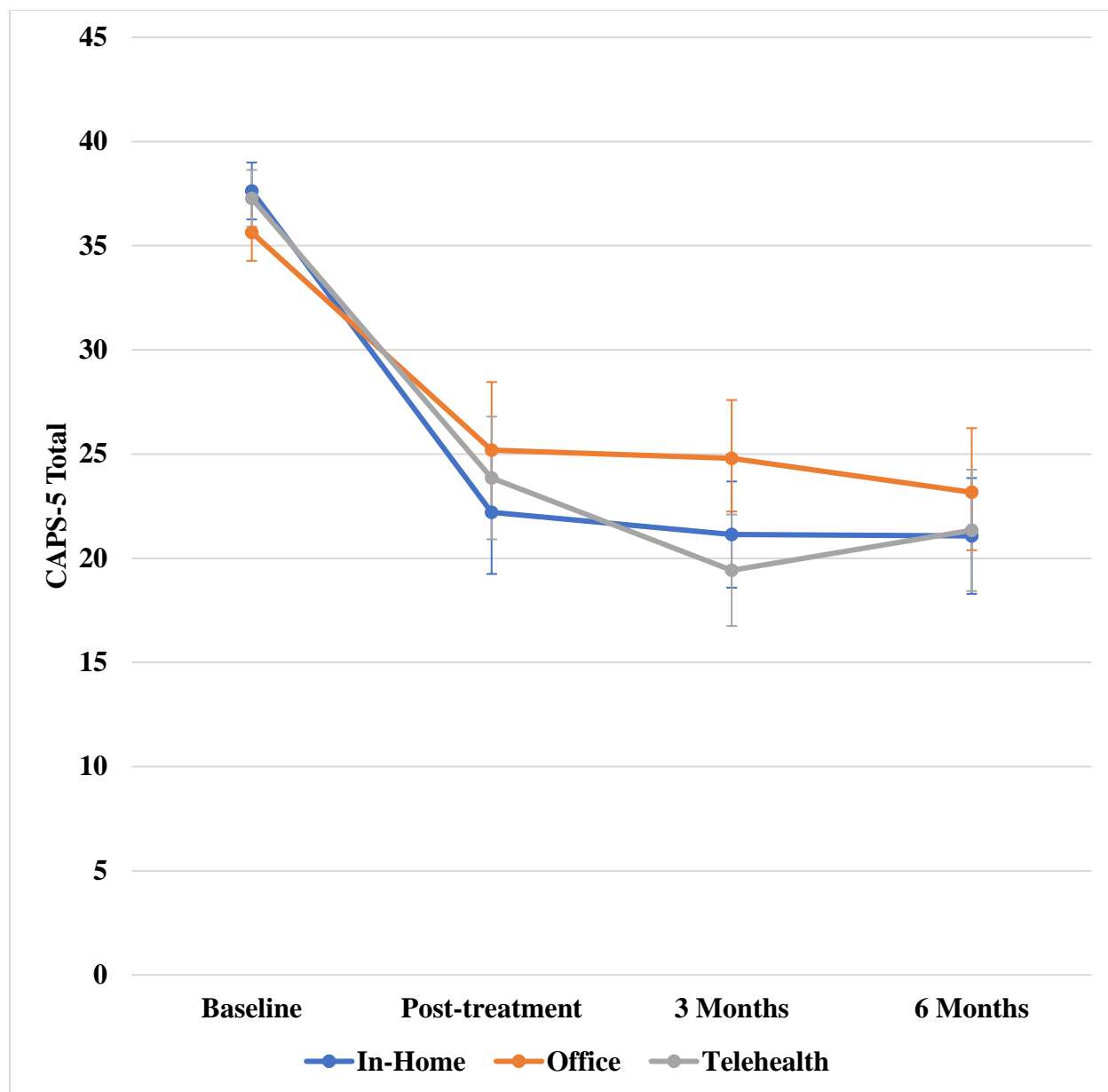

Supplement: Supplementary file 3 — Additional file 3: Supplementary Figure 3. Changes in CAPS-5 totals from baseline to 6 months posttreatment for the full sample. CAPS-5 = Clinician-Administered PTSD Scale for DSM-5. [file 12888_2022_3699_MOESM3_ESM.pdf]

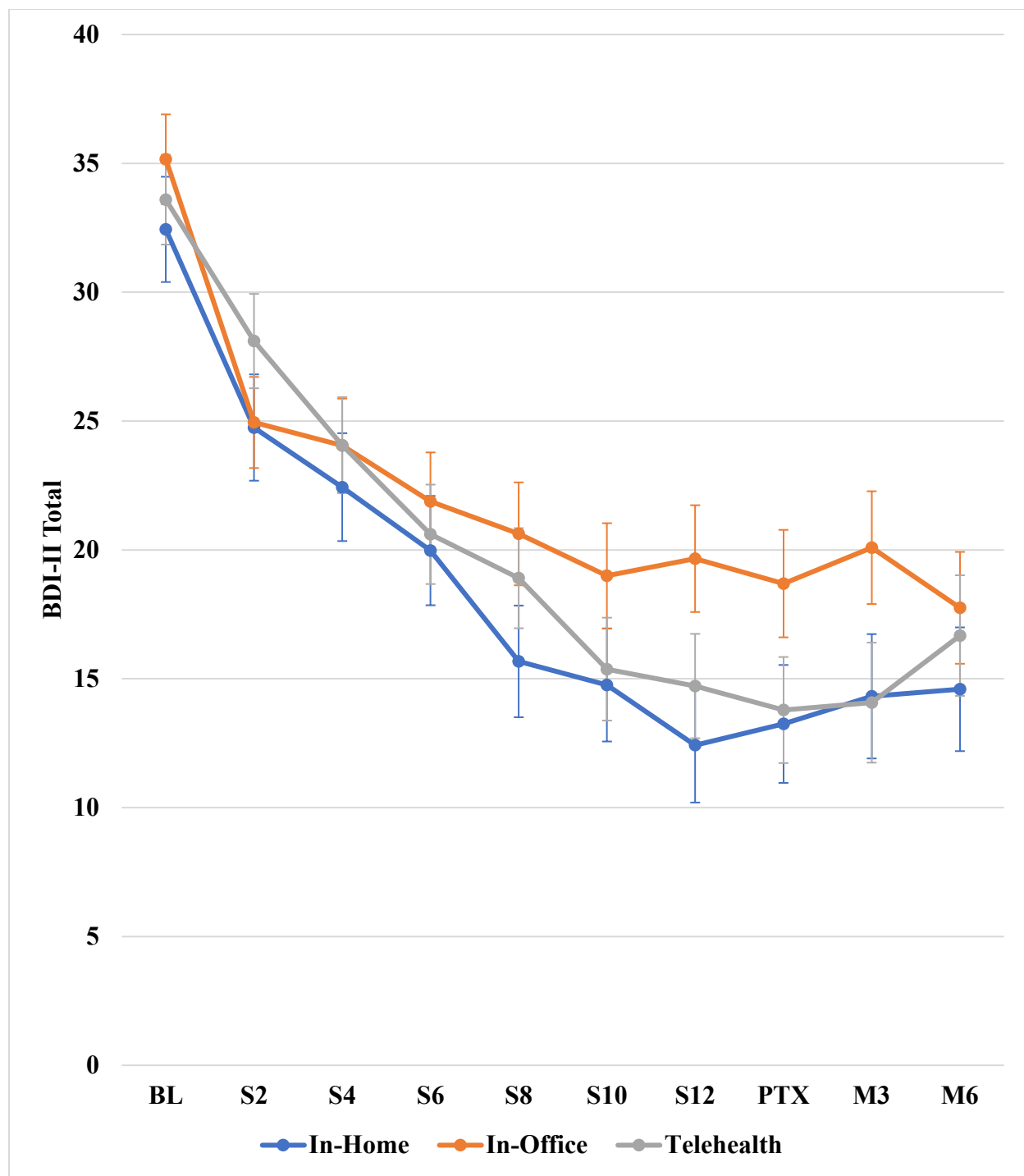

Supplement: Supplementary file 4 — Additional file 4: Supplementary Figure 4. Changes in BDI-II totals from baseline to 6 months posttreatment for the full sample. BDI-II = Beck Depression Index II; BL = baseline; M = month; PTX = posttreatment; S = session. [file 12888_2022_3699_MOESM4_ESM.pdf]
