# Supplementary material for: In-office, in-home, and telehealth cognitive processing therapy for posttraumatic stress disorder in veterans: a randomized clinical trial
Source: BMC Psychiatry. 2022 Jan 17;22:41. doi: 10.1186/s12888-022-03699-4 (PMC8763446; doi:10.1186/s12888-022-03699-4)
Supplement: Supplementary file 5 — Additional file 5: Supplementary Table 1. PTSD Checklist for DSM-5 (PCL-5) change during treatment with pairwise differences for full sample as compared with equipoise-stratified samples. [file 12888_2022_3699_MOESM5_ESM.docx]

**SUPPLEMENTARY TABLE 1.** **PTSD Checklist for *DSM-5* (PCL-5) change during treatment with pairwise differences for full sample as compared with equipoise-stratified samples**

|  | Full Sample | Equipoise Strata (Opted-out of One Arm) | | |
| --- | --- | --- | --- | --- |
|  | (All Subjects) | No Telehealth | No In-Home | No Office |
| Strata Included (N at baseline) | All (N = 120) | A, B (n = 46) | A, D (n = 71) | A, C (n = 54) |
| Telehealth | -26.6 (2.6) | N/A | -24.6 (2.8) | -29.7 (3.2) |
| In-Home | -27.8 (2.8) | -30.4 (2.8) | N/A | -28.8 (3.0) |
| Office | -17.6 (2.6) | -18.0 (3.0) | -17.8 (2.9) | N/A |
| Significance of Pairwise Differences (p values) | | | | |
| In-Home v. Office | 0.009 | 0.002 |  |  |
| Telehealth v. Office | .015 |  | 0.075 |  |
| In-Home v. Telehealth | .77 |  |  | 0.830 |
