# Supplementary material for: In-office, in-home, and telehealth cognitive processing therapy for posttraumatic stress disorder in veterans: a randomized clinical trial
Source: BMC Psychiatry. 2022 Jan 17;22:41. doi: 10.1186/s12888-022-03699-4 (PMC8763446; doi:10.1186/s12888-022-03699-4)
Supplement: Supplementary file 6 — Additional file 6: Supplementary Table 2. Change in Clinician-Administered PTSD Scale for DSM-5 (CAPS-5) from baseline to posttreatment for full-sample compared with equipoise-stratified samples. [file 12888_2022_3699_MOESM6_ESM.docx]

**SUPPLEMENTARY TABLE 2.** **Change in Clinician-Administered PTSD Scale for *DSM-5* (CAPS-5) from baseline to posttreatment for full-sample compared with equipoise-stratified samples**

|  | Full Sample  (All Subjects) | Equipoise Strata (Opted-Out of One Arm) | | |
| --- | --- | --- | --- | --- |
|  |  | No Tele | No In-Home | No Office |
| Strata included (N at baseline) | All (N = 120) | A, B (n = 46) | A, D (n = 71) | A, C (n = 54) |
| Telehealth | -13.4 (2.78) | N/A | -9.4 (2.9) | -18.2 (3.8) |
| In-Home | -15.4 (3.06) | -19.4 (3.6) | N/A | -15.9 (3.7) |
| In-Office | -10.4 (2.80) | -14.5 (3.8) | -8.9 (2.8) | N/A |
| Significance of Pairwise Differences (p values) | | | | |
| In-Home v. In-Office | 0.23 | 0.35 |  |  |
| Telehealth v. In-Office | 0.45 |  | 0.90 |  |
| In-Home v. Telehealth | 0.63 |  |  | 0.66 |
