# Supplementary material for: In-office, in-home, and telehealth cognitive processing therapy for posttraumatic stress disorder in veterans: a randomized clinical trial
Source: BMC Psychiatry. 2022 Jan 17;22:41. doi: 10.1186/s12888-022-03699-4 (PMC8763446; doi:10.1186/s12888-022-03699-4)
Supplement: Supplementary file 7 — Additional file 7: Supplementary Table 3. Change in Beck Depression Inventory, Second Edition (BDI-II) from baseline to posttreatment for full-sample as compared with equipoise-stratified samples. [file 12888_2022_3699_MOESM7_ESM.docx]

**SUPPLEMENTARY TABLE 3.** **Change in Beck Depression Inventory, Second Edition (BDI-II) from baseline to posttreatment for full-sample as compared with equipoise-stratified samples**

|  | Full Sample  (All Subjects) | Equipoise Strata (Opted-out of One Arm) | | |
| --- | --- | --- | --- | --- |
|  |  | No Tele | No In-Home | No Office |
| Strata included (N at baseline) | All (N = 120) | A, B (n = 46) | A, D (n = 71) | A, C (n = 54) |
| Telehealth | -13.5 (1.9) | N/A | -13.4 (2.0) | -16.0 (2.4) |
| In-Home | -12.4 (2.0) | -15.0 (2.2) | N/A | -12.4 (2.3) |
| Office | -5.7 (1.9) | -5.3 (2.3) | -5.8 (2.1) | N/A |
| Significance of Pairwise Differences (p values) | | | | |
| In-Home v. Office | 0.017 | 0.002 |  |  |
| Telehealth v. Office | 0.004 |  | 0.006 |  |
| In-Home v. Telehealth | 0.703 |  |  | 0.27 |
