# Supplementary material for: In-office, in-home, and telehealth cognitive processing therapy for posttraumatic stress disorder in veterans: a randomized clinical trial
Source: BMC Psychiatry. 2022 Jan 17;22:41. doi: 10.1186/s12888-022-03699-4 (PMC8763446; doi:10.1186/s12888-022-03699-4)
Supplement: Supplementary file 8 — Additional file 8: Supplemental Table 4. Number of participants who completed assessment visit during study. [file 12888_2022_3699_MOESM8_ESM.docx]

**SUPPLEMENTAL TABLE 4. Number of participants who completed assessment visit during study.**

|  | In-Home | Office | Telehealth |
| --- | --- | --- | --- |
| **Assessment Timepoint** | n | n | n |
| Baseline | 32 | 44 | 44 |
| 1-month follow-up | 16 | 21 | 19 |
| 3-month follow-up | 20 | 26 | 24 |
| 6-month follow-up | 15 | 25 | 18 |
